# International society of sports nutrition position stand: coffee and sports performance

**DOI:** 10.1080/15502783.2023.2237952

**Published:** 2023-07-27

**Authors:** Lonnie M. Lowery, Dawn E. Anderson, Kelsey F. Scanlon, Abigail Stack, Guillermo Escalante, Sara C. Campbell, Chad M. Kerksick, Michael T. Nelson, Tim N. Ziegenfuss, Trisha A. VanDusseldorp, Douglas S. Kalman, Bill I. Campbell, Richard B. Kreider, Jose Antonio

**Affiliations:** a Walsh University, Department of Exercise Science, North Canton, OH, USA; b Nutrition, Exercise and Wellness Associates, Cuyahoga Falls, USA; c Indiana Institute of Technology, Department of Biological and Physical Sciences, Fort Wayne, USA; d University of Mount Union, Department of Exercise, Nutrition, and Sport Science Alliance, USA; e California State University, Department of Kinesiology, San Bernardino, USA; f The State University of New Jersey, Department of Kinesiology and Health, Rutgers, New Brunswick, USA; g Lindenwood University, Exercise and Performance Nutrition Laboratory, St. Charles, USA; h Carrick Institute, Cape Canaveral, USA; i Center for Applied Health Sciences, Canfield, USA; j Bonafide Health, LLC p/b JDS Therapeutics, Harrison, USA; k Jacksonville University, Department of Health and Exercise Sciences, Jacksonville, USA; l Nova Southeastern University, Department of Nutrition, College of Osteopathic Medicine, Fort Lauderdale, USA; m University of South Florida, Performance & Physique Enhancement Laboratory, Tampa, USA; n Texas A&M University, Exercise & Sport Nutrition Lab, Department of Kinesiology and Sports Management, College Station, USA; o Nova Southeastern University, Department of Health and Human Performance, Davie, USA

**Keywords:** Coffee, caffeine, bioactives, chlorogenic acids, polyphenols, ergogenic

## Abstract

Based on review and critical analysis of the literature regarding the contents and physiological effects of coffee related to physical and cognitive performance conducted by experts in the field and selected members of the International Society of Sports Nutrition (ISSN), the following conclusions represent the official Position of the Society:

(1) Coffee is a complex matrix of hundreds of compounds. These are consumed with broad variability based upon serving size, bean type (e.g. common Arabica vs. Robusta), and brew method (water temperature, roasting method, grind size, time, and equipment).

(2) Coffee’s constituents, including but not limited to caffeine, have neuromuscular, antioxidant, endocrine, cognitive, and metabolic (e.g. glucose disposal and vasodilation) effects that impact exercise performance and recovery.

(3) Coffee’s physiologic effects are influenced by dose, timing, habituation to a small degree (to coffee or caffeine), nutrigenetics, and potentially by gut microbiota differences, sex, and training status.

(4) Coffee and/or its components improve performance across a temporal range of activities from reaction time, through brief power exercises, and into the aerobic time frame in most but not all studies. These broad and varied effects have been demonstrated in men (mostly) and in women, with effects that can differ from caffeine ingestion, per se. More research is needed.

(5) Optimal dosing and timing are approximately two to four cups (approximately 473–946 ml or 16–32 oz.) of typical hot-brewed or reconstituted instant coffee (depending on individual sensitivity and body size), providing a caffeine equivalent of 3–6 mg/kg (among other components such as chlorogenic acids at approximately 100–400 mg per cup) 60 min prior to exercise.

(6) Coffee has a history of controversy regarding side effects but is generally considered safe and beneficial for healthy, exercising individuals in the dose range above.

(7) Coffee can serve as a vehicle for other dietary supplements, and it can interact with nutrients in other foods.

(8) A dearth of literature exists examining coffee-specific ergogenic and recovery effects, as well as variability in the operational definition of “coffee,” making conclusions more challenging than when examining caffeine in its many other forms of delivery (capsules, energy drinks, “pre-workout” powders, gum, etc.).

## 1. Introduction

Coffee, of course, is not synonymous with liquid caffeine. Instead, it is a complex matrix of numerous compounds [1–6]. (See Table 1 for a brief summary). These compounds are consumed with broad variability based upon serving size, bean type, roasting method, brew method (e.g. water temperature, grind size, time, and equipment) and additives [1,3–9]. For example, compared to common Arabica beans, Robusta beans are typically considered higher in caffeine. Black coffee’s combination of phytochemicals in a low-calorie matrix has led researchers to place it above other foods in nutrient density rankings [10] and to deem it a part of a healthy diet, even at eight or more cups per day [11]. According to Lotfield, et al. (2018) [11] “Coffee drinking was inversely associated with mortality, including among those drinking eight or more cups per day and those with genetic polymorphisms indicating slower or faster caffeine metabolism. These findings suggest the importance of noncaffeine constituents in the coffee-mortality association and provide further reassurance that coffee drinking can be a part of a healthy diet.” The focus of this position stand is the complexity of coffee, its role in sports nutrition, and how it interacts with exercise.

**Table 1.** Select non-water components of coffee that may affect health or performance.

Component	Percent^1^	Percent, Roasted^2^	Purported Benefit
Caffeine	.5–2%	.5–1% (50–400 mg)	Alertness, reaction time, sense of energy, muscular strength and endurance
Total polyphenols (incl. CGA)	6–8%	1–2% (35–500 mg)	Blood flow, glucose disposal
Lipids	12–18% (1–2% fatty acids)	15–20% (1–2% fatty acids)	Energy source^4^
Carbohydrates	50–60%	25–40% (200–800 mg soluble fiber)	Energy source^4^, prebiotic^4^
Proteins	6–8% (1–2% amino acids)	13–15% (0% free amino acids)	Energy source^4^, growth/repair^4^
Total Minerals	3–4%	3–5% (200–700 mg)	Varies
Melanoidins^3^	–	15–20% (brown Maillard polymers)	Antioxidant, anti-inflammatory

^1^Unroasted (green) generalized coffee, typically arabica bean; ^2^Moderately roasted coffee; CGA = Chlorogenic Acid (primary polyphenol, approx. 40% of whole coffee cherry). ^3^Melanoidins are brown nitrogen-containing polymers, including acrylamide, from the Maillard browning reaction that increase with roasting. Partially sourced from Higashi, 2019. ^4^Insufficient amounts regarding exercise.

Being a part of many cultures for centuries [7], coffee has historically been researched from a variety of disciplines. Agricultural, sensory, psychometric, and physiologic aspects of coffee have been studied in parallel and sometimes in converging ways [7,12–16]. Agricultural research has focused upon such things as bean type and genetic modifications, which in turn affect nutritional content and thus application to mental and physical performance [7,9,14]. Sensory scientists have developed the World Coffee Research Sensory Lexicon [6] – a tool that helps describe perceptions as one consumes coffee, perceptions that may affect physiology, appetite, mental, and physical performance [12,17,18]. Psychometrically, coffee has been studied in areas, such as mood and cognitive performance (Table 2). Lastly, the physical outcomes of consuming coffee and its inherent ingredients – including caffeine – have captured the interest of exercise physiologists and others seeking to test and apply them to athletic performance, recovery, and fat loss [2,29,32,54,55] (See Table 2). This body of research is not large when limited to coffee itself, but has grown since early observations roughly a century ago. Contreras-Barraza et al. [56] cited the relationship between coffee/caffeine and sport as originating in 1938, though Rivers and Webber [57] published data regarding the action of caffeine on the capacity for muscular work as early as 1907. The growth and evolution of exercise physiology literature related to coffee are unsurprising given its prevalence, and data that 76% of post-competition urine samples of elite athletes contained measurable concentrations of coffee’s most well-known ingredient – caffeine [58].

**Table 2.** Summary of studies exploring the exercise-related effects of coffee.

Study	Participants	Exercise-related performance test	Coffee (caffeinated) dose	Timing of ingestion	Main findings
Alkatan, 2020 [19]	18 healthy male university students(18–25 yr)	25-meter simulated freestyle swimming competition	2 shots of coffee (250 mg caffeine), 2 shots of decaffeinated (DCF) coffee (minimal caffeine)	45 mins before exercise	Swimming time ↔
Anderson et al. 2018 [20]	9 experienced cyclists, 7 males, 2 females(37.3 ± 5.0 yr)	30-second Wingate	Via® coffee (280 mg caffeine), DCF coffee	60 min before exercise	Peak power ↔ Mean power ↔ Fatigue index ↔
Anderson et al. 2020 [21]	10 competitively trained cyclists, 9 males, 1 female (38.1 ± 6.5 yr)	30-second Wingate	Via® coffee (280 mg caffeine), DCF coffee	45 min before exercise	Peak power ↔ Mean power ↔ Power drop ↔
Araújo et al., 2015 [22]	14,563 public service workers (35–74 yr)	Standardizedneuropsychological test battery	Habitual coffee consumption (never/almost never, ≤1 cup/day, 2–3 cups/day, ≥3 cups/day) in the last 12 months	N/A	Words recalled ↑ Verbal fluency ↑
Camfield et al., 2013 [23]	60 light to moderate healthy coffee drinkers(≤16 cups/week) (≥50 yr)	Cognitive and mood assessments	6 g DCF green coffee blend (GB) (532 mg CGA/5 mg caffeine/cup), 5.7 g CGA treatment (530 mg CGA/8.8 mg caffeine/cup), 6 g maltodextrin placebo (0 g CGA/0.7 mg caffeine/cup)	40 mins before testing	CGA ↔ cognition GB ↑ sustained attn GB ↓ decision time GB ↑ alertness
Church et al., 2015 [24]	20 healthy recreationally active habitual caffeine consumers (>200 mg/day), 10 males, 10 females(24.1 ± 2.9 yr)(24.1 ± 2.9 yr) (24.1 ± 2.9 yr)	5 km run time trial, upper body (UB) and lower body reaction to visual stimuli, and multiple object tracking	200 ml Turkish coffee (3 mg/kg caffeine)	60 min before exercise	5 km time ↔ UB reaction time ↓ RER ↑
Clark et al., 2016 [25]	12 recreationally active males (20–24 yr)	18 cycle ergometer 4-second sprints with 116 seconds recovery	0.09 g/kg coffee (3 mg/kg caffeine), 3 mg/kg caffeine, taste-matched placebo, control	45 min before exercise	Peak power ↔ Mean power ↔ Mean RPE ↔
Clark et al., 2018 [26]	13 trained male runners(24 ± 6 yr)	1 mile race	0.09 g/kg coffee (3 mg/kg caffeine) (COF), 0.09 g/kg DCF coffee, placebo	60 min before exercise	COF race time ↓
Clark et al., 2019 [27]	38 adults, 19 males(30 ± 5 y), 19 females(28 ± 6 yr)	5 km cycling time trial	0.09 g/kg coffee (3 mg/kg caffeine) in 300 ml of water, placebo in 300 ml of water, control	60 min before exercise	Speed ↑
Clarke & Richardson 2021 [28]	46 recreationally active adults, 27 males, 19 females (29 ± 6 yr)	5 km cycling time trial	0.09 g/kg coffee (3 mg/kg caffeine), placebo	60 min before exercise	Speed ↑
Costill et al 1978 [29]	9 competitive cyclists, 7 males, 2 females	80% VO2max cycling trial until exhaustion	5 g DCF coffee with added 330 mg caffeine in 200 ml of hot water	60 min before exercise	RPE ↓ Lipid metabolism↑ Time ↑
Cropley et al., 2012 [30]	39 healthy older participants	Mood and cognitive processes via battery of cognitive tasks	3 cups DCF coffee high in CGA, DCF coffee with regular CGA, caffeinated coffee, or placebo	Unknown	Caffeinated coffee ↑ higher-level mood and attention DCF coffee high in chlorogenic acid ↑ mood and behavioral measures
Fidler et al 2015 (a) [17]	23 resistance trained university students(21.2 + 3.7 yr)	Explosive Smith machine bench press,Likert-type psychometric testsLikert-type psychometric tests Likert-type psychometric tests	6.6 g Via® instant coffee (328 mg caffeine) reconstituted in 525 ml tap water	60 min before exercise	Neuromuscular performance per perceptual enhancement ↔
Graham et al., 1998 [2]	9 actively training endurance runners, 8 males, 1 female(21–47 yr)	85% VO2max run until exhaustion	7.15 ml/kg (4.45 mg/kg caffeine)	60 min before exercise	Endurance ↔
Harris, et al. 2019 (a) [31]	37 university students, 19 males, 8 females	Likert-type alertness test and serum epinephrine (SE) detection via ELISA in a subset of participants (6 males, 4 females)	6.6 g Via® instant coffee (328 mg caffeine) reconstituted in 525 ml tap water	60 min before exercise	Alertness ↑ % change of SE ↑
Hodgson et al., 2013 [32]	8 trained male cyclists/triathletes (41 ± 7 yr)	30 mins steady state (SS) cycling at 55% VO2max followed by a 45 min energy-based target time trial (TT): 70% Wmax	Instant coffee (5 mg caffeine/kg), 5 mg caffeine/kg, instant DCF coffee, placebo	60 min before exercise	SS O_2_ uptake ↔ TT mean power ↑ TT time ↓
Hoffman et al., 2007 [33]	10 physically active college students, 8 males, 2 females (2.9 ± 1.7 yr)	2 30-second Wingate tests and 2 75% VO2max cycle trials to exhaustion	1.5 cups of JavaFit nutritionally enriched coffee (JF) or DCF coffee	30 min before exercise	Peak power ↔ Mean power ↔ Time to peak ↔ Fatigue index ↔ JF time ↑ EPOC ↔
Jyväkorpi et al, 2021 [34]	126 Helsinki Businessmen Study survivors (mean age 87 yr)	Short Physical Performance Battery (SPPB) test, gait speed (4-meter walk, m/s) body composition, resting pulse, cognition using The Montreal Cognitive Assessment Screening tool	3 groups according to their daily coffee consumption (1) < 110 g, (2) 110–330 g, and (3) > 330 g	Daily	Gait speed ↑ SPPB ↑ BMI ↔ WC ↔ PA ↔ Cognition ↔
Karayigit et al., 2020 [35]	17 adults (23 ± 2 yr)	Muscular endurance, cognitive tests, heart rate variability (HRV) assessments	600 ml coffee (3 mg/kg caffeine), 600 ml coffee (6 mg/kg caffeine)	Unknown	LB power ↑ LB musc. end. ↑ Cognition ↑ UB musc. end. ↔ HRV ↔
Landry et al., 2019 [36]	11 recreationally resistance trained males, (21.8 ± 1.5 yr)	21 min high intensity interval training (HIIT) cycling, 7 resistance exercises (3 sets of 10 reps, 65% 1RM), reps to fatigue and soreness rating 24 hr later, serum testosterone (ST) assessment	355 ml coffee (144 mg caffeine) + anhydrous caffeine pill total (6 mg/kg caffeine)	60 min before exercise	ST 30 min post exercise ↑ ST response to exercise ↔ Chest press reps to fatigue ↔ Leg press reps to fatigue ↔ Soreness ↔
Leelarungrayub et al., 2011 [37]	16 healthy sedentary men (20–21 yr)	Modified Bruce VO2max test with RER and HR analysis at 80% maximal HR	Caffeinated coffee, (ml used for reconstitution unknown) (5 mg/kg caffeine)	60 min before exercise	VO_2_ ↑ RER ↓ Glucose post exercise ↑ Lipid peroxide levels ↑ Antioxidant capacity ↔
Marques et al. 2018 [38]	12 healthy experienced male runners (23.5 ± 3.9 yr)	800-meter run	Soluble coffee (5.5 mg/kg caffeine) dissolved in 200 ml hot water, DCF	60 min before exercise	Time ↔ RPE ↔
Mohney et al. 2015 (a) [39]	Caffeine and non-caffeine habituated resistance trained university students (21.2 + 3.7 yr)	Explosive Smith machine bench press	6.6 g Via® instant coffee (328 mg caffeine) reconstituted in 525 ml tap water	60 min before exercise	Peak force ↑ Peak power ↑ Peak velocity ↑ Rate of force development ↑
Mohney et al 2018 (a) [40]	14 university baseball/softball athletes(18–25 yr)	Pitch speed, accuracy, consistency	6.6 g Via® instant coffee (328 mg caffeine) reconstituted in 525 ml tap water	60 min before exercise	Ball velocity ↑ Pitch accuracy ↓ (trend) Pitch consistency ↔
Nieman et al., 2018 [41]	15 adult cyclists	50 km cycling time trial	300 ml/day of high chlorogenic acid coffee (1,066 mg CGA, 474 mg caffeine)	2 weeks	50 km time ↔ 50 km power ↔ HR post 30 mins ↑ VO_2_ post 30 mins ↑
Powers et al. 2013 (a) [42]	12 resistance trained adults (18–35 yr)	50% 1 RM bench press	6.6 g Via® instant coffee (328 mg caffeine) reconstituted in 525 ml tap water	60 min before exercise	Peak force ↑ Peak power ↑ Rate of force development ↑
Reed et al., 2019 [43]	30 healthy adults(18–49 yr)	Series of cognitive tasks and motivation assessment	300 mg coffeeberry extracts, 100 mg coffeeberry extracts, placebo, 75 mg caffeine (positive control)	60 min before tasks	Cognition ↔ Alertness ↑ Motivation ↔ Fatigue perception ↓
Richardson & Clarke, 2016 [44]	9 resistance trained males (22–26 yr)	60% 1RM squat and bench press until failure	0.15 g/kg caffeinated coffee in 600 ml of hot water, 0.15 g/kg DCF, 0.15 g/kg DCF +5 mg/kg anhydrous caffeine, 5 mg/kg anhydrous caffeine, placebo	45 min before exercise	Squat reps ↑ Bench reps ↔ Squat, total weight lifted ↔ Bench, total weight lifted ↑ RPE squat ↔ RPE bench ↔
Ruffner et al., 2018 (a) [45]	34 resistance trained adults (18–35 yr)	50% 1 RM reflexive (myotatic) bench press	6.6 g Via® instant coffee (328 mg caffeine) reconstituted in 525 ml tap water	60 min before exercise	Myotatic peak force ↑ Myotatic time to peak power ↓
Sherman (2016) [46]	83 university students who consumed ≥ a moderate amount of caffeine/week (18–21 yr)	Computer-based memory and word identification assessment, 1–5 scales for wakefulness	Coffee (180 mg caffeine), DCF coffee (7–10 mg caffeine)	Matched via age, MEQ scores, and reported caffeine use	Early morning memory ↑ Afternoon wakefulness ↑
Slack et al. 2016 (a) [47]	34 university students	Self-selected, maximal effort, reflexive Smith machine bench press (i.e. preceded by prior stretch)	6.6 g Via® instant coffee (328 mg caffeine) reconstituted in 525 ml tap water, DCF coffee, H_2_O	60 min before exercise	Myotatic force (trend) ↑ Myotatic time to peak power (trend) ↓ Myotatic power ↑ Myotatic velocity ↑
Smith 2016 (a) [48]	10 resistance trained university students	Likert-type psychometric tests; 50% 1 RM bench press	6.6 g Via® instant coffee (328 mg caffeine) reconstituted in 525 ml tap water, DCF coffee, H_2_O	60 min before exercise	Alertness ↑ Focus ↔ Peak power ↑ Peak velocity ↑
Smith 2013 [49]	128 adults, 64 males, 64 females (18–65 yr)	Cognitive function assessments	Coffee (65 mg caffeine), DCF coffee	Unknown	Processing speed↑ Memory ↔
Soga et al. (2013) [50]	18 healthy males(36.1 ± 7.4 yr)	Energy expenditure (EE) via indirect calorimetry	329 mg CGA (50 mg caffeine) daily for 4 weeks	1 185 ml can/day within 1 hr during daytime	Postprandial EE ↑ Postprandial fat utilization ↑
Trexler et al., 2016 [51]	54 resistance trained males	Leg (LP) and bench press (BP) 1RM, 80% 1RM repetitions to failure (RTF), 5 10 s cycle ergometer sprints	8.9 g coffee (303 mg caffeine)	30 min before exercise	LP 1 RM ↑ LP RTF ↔ BP 1RM ↔ BP RTF ↔ Sprint PP ↔ Sprint TW ↔ Repeated sprint fatigue ↓
White et al 201 8 (a) [52]	9 resistance trained university students	6 reps at 50% 1 RM for bench and squat, 6 maximal vertical jump repetitions, serum epinephrine (SE) detection via ELISA	6.6 g Via® instant coffee (328 mg caffeine) reconstituted in 525 ml tap water, DCF coffee, H_2_O	60 min before exercise	SE (trend) ↑
Wise et al., 2014 (a) [53]	23 resistance trained university students	50% 1RM bench press	6.6 g Via® instant coffee (328 mg caffeine) reconstituted in 525 ml tap water	60 min before exercise	Peak force ↑ Peak power ↑ Peak velocity ↑ Rate of force development ↑

↑= significant increase; ↓= significant decrease; ↔ = no significant difference; (a) = abstract only; BP = bench press; CGA = total chlorogenic acids; COF = caffeinated coffee, DCF = decaffeinated coffee; EE = energy expenditure; GB = green coffee blend; HIIT = high intensity interval training; H_2_O = water control; HRV = heart rate variability; LB = lower body; LP = leg press; MEQ = morningness-eveningness questionnaire; PA = physical activity; RTF = repetitions to failure; SE = serum epinephrine; SPPB = short physical performance battery; SS = steady state; ST = serum testosterone; TT = time trial; UB = upper body; WC = waist circumference.

A final introductory point that should be noted upon reading this position stand is that there is necessary repetition and referral among sections. The complex interrelationships between coffee, caffeine, physiology, and research methodology call for it. Every attempt has been made to minimize redundancy and an excessive focus on caffeine proper, which is the purpose of a different ISSN position stand [59].

## 2. Methods

ISSN Position Stands are invited papers of topics the ISSN Editors and Research Council identify as topics of interest to our readers who need Position Stands to provide guidance to readers and the profession. Editors and/or the Research Committee identify a lead author or team of authors to perform a comprehensive literature review. The draft is then sent to leading scholars for review and comment. The paper is then revised as a consensus statement and reviewed and approved by the Research Committee and Editors as the official position of the ISSN.

A review of the scientific literature was conducted related to the physiological effects and nutritional aspects of coffee with keywords including history, serving size, bean type, brew method, sensory, psychometric, chlorogenic acids, antioxidant, endocrine, neuromuscular, cognitive, metabolic (e.g. glucose disposal and vasodilation), hydration, exercise performance, exercise recovery, dose, timing, absorption, habituation, nutrigenetics, microbiota, sex, training status, reaction time, power exercises, aerobic exercise, side-effects, and dietary interactions. This was accomplished by conducting keyword searches related to coffee using the National Institutes for Health National Library of Medicine PubMed.gov search engine.

## 3. History of research on coffee related to exercise

According to Nieber [60], coffee’s origins reach back to the 1100s. Although coffee as a beverage has changed in form since the coffee fruit was discovered in the Ethiopian mountains, it spread through Arabia, and then, in the 1600s [61] to Europe. Interestingly, Chou (1992) [62], citing legend, reported its discovery in “Abyssinia (Upper Egypt)” as early as 850 [63]. It is unlikely that taste alone was the cause of coffee’s spread; coffee’s caffeine content and “entourage effect” with other inherent components elicit an obvious stimulatory state [13,15,16,23,64]. In the modern scientific era, exercise physiologists have borrowed from multiple lines of inquiry (e.g. agricultural, metabolic, sensory, and psychometric), to test coffee and its components in various ways. As with many nutrients and dietary supplements, the effects have varied depending on many factors (see section titled: Control issues in coffee research and practical application and Table 2).

### 3.1. Defining a cup of coffee (relevant to exercise)

A seemingly simple descriptor, “one cup,” can be complex when an operational definition is sought for research purposes. First, volume needs to be considered. To a barista or coffee aficionado, a serving of espresso is typically one fluid ounce (29.6 ml, 28 g) and per the Specialty Coffee Association Heritage Cupping Standards, approximately five fluid ounces (150 ml) of coffee in a seven-to-nine-ounce (207–266 ml) vessel is appropriate [65]. In standard kitchen applications, a cup (of coffee) is eight fluid ounces (237 ml). For a nutritionist or food scientist, a cup of coffee is 248 g per the USDA Food Data Central database [66]. In commercial settings, a mid-sized “grande” coffee may be 16 fluid ounces (474 ml). Second, when holding volume constant, the composition within the volume of one serving needs to be addressed. Flavorings and additives affect how much brewed coffee is in a cup and, as previously stated, bean type, roasting, grind size, and brew method affect which coffee constituents are able to be identified therein. Subsequently, researchers have addressed the wide range of bioactives in “a cup” of served coffee, including its relevance to exercise [2,4,5,9,32,67]. These discrepancies leave researchers defining a cup of coffee in different ways in their interventions. Two seminal exercise physiology studies provide examples.

Early work by Costill et al. [29] used an intervention of brewed decaffeinated coffee and a caffeinated (330 mg) equivalent with nine competitive cyclists (seven males, two females) who exercised until exhaustion on a bicycle ergometer at 80% of VO_2_ max. The coffee was served as 200 ml hot water containing 5 g decaffeinated or caffeinated coffee, the latter described as containing a “quantity of caffeine commonly consumed in 2.5 cups of regularly percolated coffee.” The ingestion, 60 min prior to performance, resulted in caffeinated coffee consumers performing an average of 90.2 (SE ±7.2) min of cycling versus an average of 75.5 (SE ±5.1) min in the decaffeinated coffee trial (*p* < 0.05). Simultaneously, the caffeinated coffee intervention induced a rise in plasma-free fatty acids and glycerol, with a concomitant decrease in respiratory exchange ratios, which led the authors to conclude that lipolytic effects (fuel provision from fat) coupled with nerve impulse enhancement likely provided the ergogenic effect. This proposed mechanism whereby coffee/caffeine enhances fat oxidation and spares muscle glycogen to enhance performance has had very little data to support it [68]; nonetheless, this landmark study has been cited 1171 times according to Google Scholar (April 2023). There was no true control (e.g. water condition), so conclusions as to whether non-caffeine coffee components affected exercise cannot be made.

Later, Graham and colleagues [2] noted that it is important to understand that there are hundreds of compounds in coffee dissolved along with caffeine and the other methylxanthines – including lipids, carbohydrates, and proteins. The investigators pointed out that these non-caffeine components represent over 60% of the compounds, whereas caffeine is approximately 2%. With this in mind, they mixed a large quantity of ground coffee well to ensure uniformity and drew repeatedly across trials from this supply. They stated: “In preliminary tests we prepared drip-filtered coffee as concentrated as the laboratory workers could tolerate with regard to taste and still consume approximately two ‘coffee mugs’ (total of 500–600 ml) in 10 min.” and “The coffee was always prepared in the same fashion (40 g ground coffee and 1,000 ml of water)” with the volume consumed based on the weight of the subject. Sixty minutes prior to exhaustive exercise, the researchers provided nine young adults (21–47 yr) who were actively trained endurance runners (eight males, one female) with various combinations of coffee and caffeine over five trials: Decaffeinated coffee; decaffeinated coffee plus caffeine; regular coffee; placebo capsules; and caffeine capsules. The authors concluded that across caffeine-containing trials (each 4.45 mg/kg), plasma caffeine and paraxanthine concentrations were similar. They also noted that after 1 h of rest, plasma epinephrine was increased (*p* < 0.05) by caffeine ingestion, with the increase being ~ 50% greater (*p* < 0.05) with caffeine capsules than with coffee. Exercise abolished these differences in epinephrine concentrations among the three caffeine trials, and the epinephrine values were all greater (*p* < 0.05) than in the other tests. Endurance was only increased (*p* < 0.05) in the caffeine capsule trial. The researchers further concluded that one cannot extrapolate the effects of caffeine to coffee and that there must be a component(s) of coffee that moderates the actions of caffeine.

### 3.2. Defining the time spectrum of physical performance as it relates to coffee

Other than attention, spatial/working memory and executive function – all intrinsic to peak performance in many sports [69] (see Table 2) – physical performance can be compartmentalized temporally. Exercise physiology textbooks have long categorized types of exercise according to the evocation of biological energy systems along a timeline [70–73]. These range from “immediate” systems that rely on very limited substrates such as phosphagens (creatine phosphate and ATP) over a few seconds, to moderately limited substrates such as glycogen that supply the most power over 1 to 2 min, to much less limited and lower power substrates such as lipids that can supply energy for hours [71–73]. It is important to define types of exercise in this manner as coffee influences mechanisms of each in ways that have impacted the hypotheses behind its ergogenic effect for researchers.

Perhaps, the briefest practical example of neuromuscular performance is simple reaction time (or response time) analysis, which can be tested in sports-specific ways or with visual or auditory cues and the press of a button on a computer keyboard [74]. Far from being dependent on complex biochemical pathways that tap stored cellular substrates such as glycogen or lipid, reaction time is nonetheless important to sport [74]. This type of physical performance is proportionately more reliant on motor behavior and sarcoplasmic calcium release and, as stated, phosphagen hydrolysis [75]. Further down the temporal and energy system spectrum are brief, high-intensity performances, such as one to four repetition maximums (1RM to 4RM), corresponding to 90–100% of maximal ability; they typically include compound (multi-joint) resistance exercise movements like the squat, deadlift, or bench press and still rely heavily on the phosphagen system [70]. Similarly, ballistic performances such as the vertical jump, shot put, snatch, or maximal dynamic efforts with barbells that employ the myotatic (stretch) reflex last only a few seconds and rely on very limited reserves of creatine phosphate and ATP [70]. Still further into a temporal timeline are popular cycle ergometer sprints such as the 30-s Wingate. Although still “anaerobic” in nature, they increasingly employ glycogenolysis. It is important for exercise physiologists and sports nutritionists not to over-rely on the 30-s Wingate as a gold standard “anaerobic” test because of the cellular energy dependence on more enzymatic steps than immediate (≤5 s) ballistic exercises. Lastly in the timeline are oxidative systems, in which glycogenolysis and beta oxidation interact with the citric acid cycle and electron transport system. Being slower and less powerful, but with the ability to underwrite much longer endurance performances, oxidative systems are impacted in different ways by coffee [29,50,76]. See Figure 2.

## 4. Coffee absorption and transport

Being a complex matrix of compounds, coffee’s constituent ingredients are absorbed and transported in different ways. For example, depending on route of administration (e.g. gum vs. capsule vs. drink), caffeine has been shown to be absorbed buccally, through the stomach, and mostly via the small intestine [59]. General absorption speed being gum > drink > capsule [59,77]. This, combined with near-100% bioavailability, leads to detection in the serum within minutes and an established, but individually variable, half-life of ~ 4–6 h [78]. In some contrast, chlorogenic acids and their metabolites are likely absorbed tri-phasically, in the stomach, in the small intestine (approximately one-third), and later in the large intestines after interactions with the local microbiota [1,79–81]. Bioavailability of intact polyphenols such as native chlorogenic acid has been described as “low” and “to a small extent,” with only 5%–10% of the total intake of dietary polyphenols absorbed through the stomach and the small intestine [1,82] but conversion to a number of metabolites [1,81] confounds the interpretation. Genetic and gut microbiota variance among individuals are also an issue [1]. Finally, intestinal absorption of (tap) water, such as that used to brew the coffee, is widely understood and hydration guidelines are commonplace. There are no conclusive data to suggest coffee, caffeinated or not, is inadequate for hydration [83]. Misinterpretation of caffeine’s diuretic effects has potential to confuse some lay persons and healthcare professionals; caffeine ingestion prior to exercise does not lead to dehydration [59,84]. Indeed, in a meta-analysis on the topic, Zhang and colleagues [84] concluded that exercise negated the minor diuretic effect of caffeine. Thus, the concerns regarding excess fluid loss due to caffeine consumption are unwarranted, particularly if the ingestion is prior to exercise. This conclusion is strengthened by the fact that coffee is typically just 1–2% caffeine and brewed coffee is largely water.

## 5. Mechanisms for the ergogenic effect of coffee

**Figure 1.**
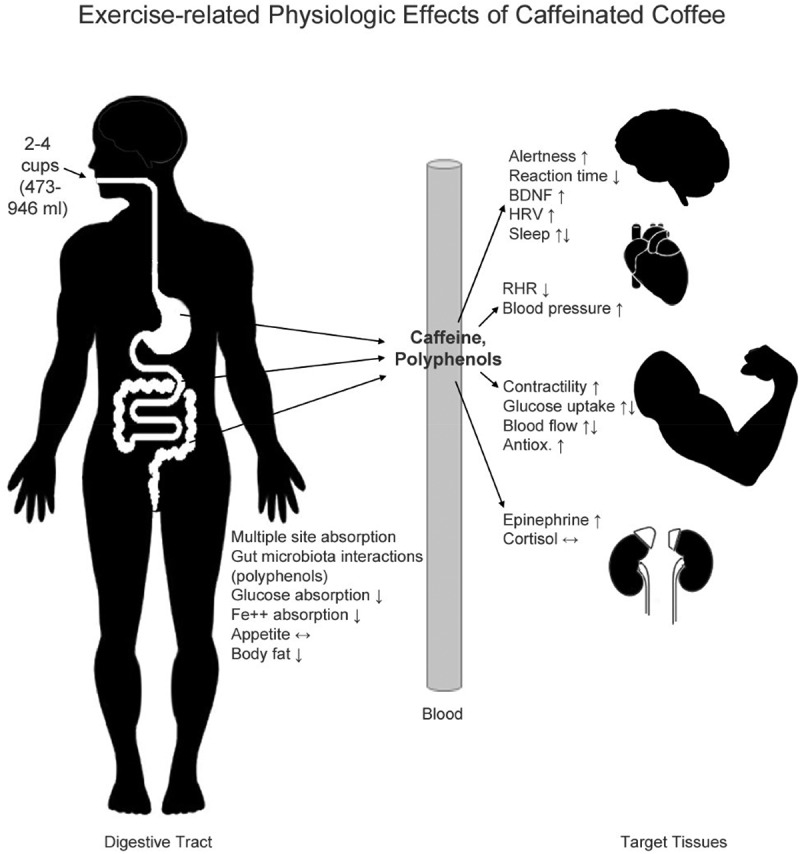
Overview of the mechanism of caffeinated coffee components’ absorption in the gastro-intestinal tract and the impact of caffeine and polyphenol (e.g. CGA) ingestion on physiology. BP = blood pressure; BDNF = brain-derived neurotropic factor; Fe^++^ = iron; HRV = heart rate variability; RHR = resting heart rate. Appearance of both up and down arrows indicates differential effects of caffeine versus polyphenols.

As noted earlier in this position stand and elsewhere, components of coffee include caffeine, chlorogenic acids, ferulic acid, caffeic acid, nicotinic acid, and unidentifiable compounds [85]. The concentrations of these components may be altered by the specific coffee variety, source of the beans, washing/drying procedures, roasting methods, storage methods, particle size of the coffee beans, and preparation practices [41,85]. An analysis of 20 espresso coffees revealed that levels of caffeine and chlorogenic acids varied significantly. Caffeine levels ranged from 51 mg/serving (Starbucks) to 322 mg/serving (Pattiserie Francoise) while chlorogenic acid levels ranged from 24 mg/serving (Starbucks) to 422 mg/serving (Pattiserie Francoise) [85]. The wide ranges of these components may impact the ergogenic effects of the beverage. See Figure 1 for a depiction of the exercise-related physiologic effects of caffeinated coffee. Chlorogenic acids have been proposed to antagonize the physiological responses of caffeine [2]. In vitro, chlorogenic acids have been shown to antagonize the adenosine receptor binding of caffeine [86], as well as to alleviate oxidative stress and inflammation. The level of chlorogenic acid derivatives found in coffee beans and their potential interference with the binding of caffeine to the adenosine receptors may be variable among coffee brands [86]. According to Hodgson et al. [32], the low concentrations of chlorogenic acids typically measured in vivo do not negatively impact the mechanisms of action of caffeine.

### 5.1. Neural (psychometric and motor)

The primary mechanism for the ergogenic effects of coffee appears to be from caffeine’s antagonism of adenosine A1 and A2A receptors [87–94]. The antagonism of adenosine receptors leads to an increase in neurotransmitter release and motor unit firing rates, pain suppression, reduced fatigue, and improved muscular performance [89,95]. Acute caffeine ingestion modifies perceptual responses that may alter performance by blocking central and peripheral adenosine receptors that influence pain signaling, and thus result in decreased pain perception at the muscle during high intensity exercise [96–99]. It has also been suggested that caffeine lowers the threshold for exercise-induced ß-endorphin release [100] which likely contributes to the impact on rating of perceived exertion. Additionally, it has been speculated that motor effects and motivational aspects are influenced when adenosine receptors are blocked through caffeine, creating a greater dopaminergic drive, and thus enhancing performance [101].

In a meta-analysis, Doherty & Smith [97] concluded that 33% of the observed improved performance was due to decreased ratings of perceived exertion (RPE) following caffeine ingestion. They also speculated that the RPE assessment may not apply to the high-intensity intermittent nature of team sports or activities such as sprinting and/or resistance exercise [97]. This conclusion is consistent with the lack of impact of coffee consumption on RPE for researchers utilizing an 800-m run [38], cycling sprint/time trial [25,27,32,102], or resistance exercise [44].

### 5.2. Endocrine, metabolic (e.g. catecholamines, glycogenolysis/lipolysis)

A number of researchers have examined the effects of caffeine on acute glucose homeostasis. Some have found caffeine to have a hyperglycemic effect [90,103] as a result of increased catecholamine release from the adrenal medulla [104–106]. The catecholamines, primarily epinephrine, bind to receptors on the membranes of skeletal muscle cells, which activate adenylate cyclase. This results in an increase in intracellular cAMP, which activates protein kinase, and accelerates the transformation of phosphorylase-b into the active form of phosphorylase-a which acts to increase the rate of skeletal muscle glycogenolysis [107]. Whether coffee or caffeine is ingested does not appear to impact the levels of epinephrine. Graham et al. [2] found a similar increase in epinephrine in response to a 4.45 mg/kg dose of caffeine ingested either in a capsule, decaffeinated coffee with added caffeine, or regular coffee.

As noted earlier, the seminal work by Costill et al. [29] proposed the increased mobilization of free fatty acids as a result of caffeine intake. The increased levels of free fatty acids were believed to result in increased oxidation of free fatty acids and sparing of muscle glycogen, based on observed increased glycerol levels and decreased RER values. However, other researchers have not found significantly lower RER values [24,108–111], increased fatty acid oxidation, nor sparing of muscle glycogen after coffee or caffeine ingestion [68,100,109,112,113]. Graham et al. [114] reexamined the prospects of caffeine sparing of muscle glycogen by pooling data from previous studies with subjects ingesting 5 or 9 mg caffeine/kg, exercising at 75–85% VO_2_max, and the inclusion of muscle glycogen measurements. Their analysis did not reveal a difference in muscle glycogen use between the caffeine and placebo trials [114]. While investigating the metabolic effects of caffeine and coffee, Hodgson et al. [32] found no differences in carbohydrate or fat oxidation during 30 min of steady-state cycling with either anhydrous caffeine (5 mg/kg), coffee (5 mg caffeine/kg), decaffeinated coffee, or placebo ingestion. They stated that this was likely due to other compounds in coffee that had subtle effects on the antagonism of adenosine receptors [32].

### 5.3. Muscular

Caffeine has been found to alter the release [91,115] or uptake [115] of calcium by the sarcoplasmic reticulum, which plays an integral role in skeletal muscle contraction. It has also been shown to inhibit phosphodiesterase (PDE), an enzyme that breaks down or reduces cAMP levels, which leads to an accumulation of cyclic adenosine monophosphate (cAMP) [91,94,116,117]. Although there is support for these mechanisms, the required concentration of caffeine (1000 µM) cannot be easily reached [94,118] as ingestion of two to three cups of coffee only results in plasma caffeine concentrations of 20–50 µM [94]. Additionally, plasma caffeine concentrations above 200 µM are associated with toxic effects and fatal poisoning is associated with concentrations above 500 µM [94]. Thus, neither of these mechanisms are likely to occur *in vivo*.

It has also been suggested that caffeine may affect the periphery via increased sodium/potassium (Na+/K+) pump activation. According to Schneiker et al. [119] caffeine’s enhancement of Na+/K+ pump activation in skeletal muscle may improve fatigue resistance. However, Paton et al. [120] speculated that caffeine may actually negatively impact fatigue resistance for repeated exercise. One reason for the discrepancy may be that a minimum dose of caffeine (>6 mg/kg) is suspected to be necessary for fatigue resistance [121].

## 6. Effects of coffee on reaction time, single-bout ballistic and power performance

### 6.1. Effects of coffee on reaction time

Reaction (response) time is the briefest performance variable likely to be affected by coffee/caffeine and is widely understood to affect sports performance [74,122]. It includes alertness to sensory stimuli, neural processing, and motor components that have been studied in various ways and in various populations. Due to a dearth of data specific to exercise-specific reaction times, several populations are presented here.

In a double-blind, placebo-controlled experiment, Smith [49] randomly provided decaffeinated or caffeinated coffee (65 mg caffeine) to 128 participants (18–65 yr, 64 males and 64 females). The results showed that relatively low-dose caffeinated coffee improved simple reaction time and the speed of encoding of new information. Given this finding, one might conclude that caffeine was causal; however, the interpretation that caffeine is solely responsible for reaction time enhancement is challenged to some extent by research on very-low-dose-caffeine coffee interventions.

For example, Robinson and colleagues [64] compared 100 mg of whole coffee cherry extract (WCCE, 1.8 mg caffeine) to a placebo (100 mg capsule of micro-cellulose) using an acute, randomized, double-blind, within-subject design using 71 participants (55–65 yr, 31 males and 40 females) with “subjective cognitive impairment,” an intermediate stage of decline between the expected changes of aging and dementia. They observed that polyphenol-rich WCCE was associated with decreased reaction time and increased brain-derived neurotropic factor (BDNF) as well as altered neuroimaging scans. Researchers concluded such acute neurophysiological changes were supportive of faster reaction times as well as sustained attention. Although suggestive, applicability to young, healthy athletes remains to be elucidated.

As noted, reaction time has cognitive processing aspects as well as motor components. Schuster and Mitchel [16] explored compounds such as catechol, pyrogallol, eicosanoyl-5-hydroxytrypamide, and chlorogenic acid as potentially enhancing brain function by upregulating dopamine and calcium release (see also [123]). Goldstein and colleagues [124] supported this conclusion, reporting increased epinephrine and dopamine in the circulation of resting healthy volunteers after ingesting two cups of either caffeinated or decaffeinated coffee.

Early clinical demonstrations that non-xanthine coffee components affect brain function appear to support such mechanistic research. Cropley et al. [30] studied 39 coffee-habituated healthy older adults who completed a series of cognitive and mood tests before and 40 min after consuming beverages that varied in caffeine and chlorogenic acid content: caffeinated coffee (167 mg caffeine, 244 mg chlorogenic acids), decaffeinated coffee (5 mg caffeine, 224 mg chlorogenic acids), high-chlorogenic acid decaffeinated green coffee (11 mg caffeine, 521 mg chlorogenic acids), or a placebo (no caffeine or chlorogenic acid). Compared to typical decaffeinated coffee, the decaffeinated (high CGA) green coffee intervention resulted in lower feelings of fatigue that stemmed from the cognitive testing session and greater feelings of alertness [30].

This conclusion was refined by Camfield et al. [23] who found the enhancement did not extend to isolated chlorogenic acid. Researchers from the same group as Cropley, et al. [30] confirmed the alertness and anti-fatigue effects observed after drinking the decaffeinated green coffee blend. However, 58 habituated older adults (>50 yr) did not experience benefits from consuming 530 mg chlorogenic acids alone. It was concluded that other coffee ingredients (or perhaps interactions in the coffee matrix) in the decaffeinated beverage were likely responsible for the favorable alertness outcome. One possibility is that quinides produced in the process of roasting green coffee beans may be involved [23,43]. It should be noted that data stemming from Cropley, et al. [30] and Camfield, et al. [23] included older participants and such findings may not be applicable to young athletes.

Other researchers, using younger populations, have also reported trends or significant improvements in alertness with decaffeinated coffee compared to water [48] or a placebo beverage [125]. This raises a concern over the use of decaffeinated coffee as a placebo, due to its bioactive aspects, as a reliable and appropriate placebo for caffeinated coffee. Collectively, these limited findings suggest an entourage effect whereby a collection of compounds in coffee work together to support cognition and reaction (response) time. Further research is needed to explicate the many possible interactions and how they might translate to exercise performance in various age groups.

### 6.2. Effects of coffee on brief ballistic performance

Knowing that the exercise performance spectrum can be broken down into multiple, time-dependent phases within the anaerobic spectrum before aerobic metabolism predominates, one might question whether any of these phases are impacted by coffee/caffeine. A temporal step beyond reaction time is brief ballistic performance. These rapid movements against a resistance (e.g. bench throws and box jumps) incorporate additional demands such as greater application of force and motor coordination than simple response time.

Unlike caffeine per se, a dearth of studies exists specific to coffee with regard to explosive power exercise. Even its more broadly studied component, caffeine, has been applied more often to endurance performance than to brief muscular strength and muscular endurance [126]. Some data do exist, however. For example, in a series of investigations published only as abstracts, and using instant coffee as a pre-exercise intervention, experienced resistance trainers (>2 yr) were tested with bench press and squat exercises. This was done after consuming two packets (6.6 g) instant/micro-ground coffee in 525 ml hot tap water [17,39,42,45,47,48,127]. Participants were given 30 min to steadily finish the beverages. The caffeinated interventions contained a total of 328 mg caffeine (placing participants in the middle of a 3–6 mg caffeine/kg ergogenic range) and the decaffeinated interventions contained <9 mg caffeine. Instant coffee was chosen for consistency in caffeine delivery (SD ±6 mg caffeinated and decaffeinated below the 9 mg calibration range) [128]. Hot tap water alone was also employed as a true control (i.e. no polyphenols) in one investigation that measured psychometric variables [48]. Instant coffee administered 60 min prior to exercise (90 min after first ingestion and 60 min after final swallow) consistently enhanced bar velocity and peak power in ballistic Smith bench press using 50% 1RM (*p* < 0.05). This effect was apparent in static, pause-and-go bench pressing, and in a three-repetition cadence, invoking the myotatic reflex [39,42,45,47,48]. The ergogenic effect did not differ between sexes when adjusted for body mass [53], although females tended to experience greater epinephrine concentrations and alertness compered to males (*p* < 0.10) [31]. Explosive performance enhancement was quantitatively lower but still statistically significant in caffeine-habituated participants [39], an effect supported by other researchers using caffeine (6 mg/kg) and countermovement jumps [129]. Similarly, Watson et al. (2002) [130] provided non-exercising caffeine-habituated and caffeine-naïve healthy volunteers a 200 mg caffeine challenge and concluded that the central and peripheral effects of tolerance are incomplete. In the series of investigations noted above, with trained individuals, brief ballistic Smith machine squatting was less enhanced than with Smith bench pressing [127]. In contrast, a repetitions-to-failure bench press and squat protocol employed by Richardson and Clarke (2016) [44] resulted in increased total work in the squat but not the bench press. More research on upper-versus-lower body exercise and on ballistic versus muscular endurance exercise is needed.

### 6.3. Effects of coffee on “substrate-dependent” muscular power, strength, and endurance

**Figure 2.**
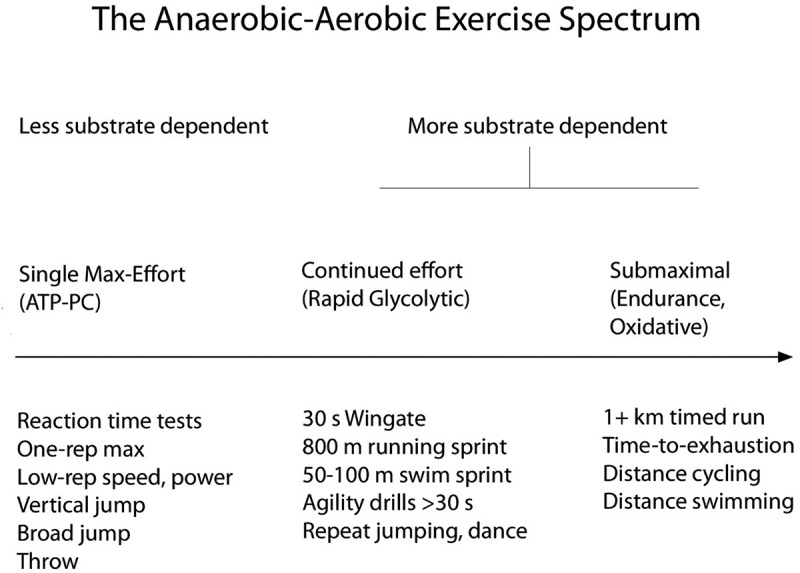
Schematic representation of the exercise spectrum affected by coffee and its components.

Beyond brief ballistic efforts, anaerobic performance can take place in more time-intensive ways (refer to Figure 2). Single, uninterrupted anaerobic efforts such as 30-s Wingate cycle sprints and swim sprints are ways to extend and examine the duration of muscular exercise. Additionally, multiple sets of anaerobic efforts extend the timeline further. Energy systems that support exercise are all substrate-dependent, including use of the immediate phosphagen (ATP-creatine phosphate) pool for perhaps 10 s. This is also true of power performance beyond 10 s – such as during a Wingate cycling test or throughout the duration of a resistance exercise session, which relies on carbohydrate metabolism [131,132] to re-phosphorylate phosphagens and engage the muscular endurance energetic pathways.

Regarding Wingate cycling performance, data on the specific effects of coffee, as opposed to caffeine, are again very limited. Hoffman et al. [33] studied 10 (eight males, two females) physically active college students, using two 30-s Wingate power tests and 1.5 cups (354 ml) of JavaFit nutritionally enriched coffee (containing 450 mg of caffeine, 1,200 mg of G. cambogia (50% hydroxycitric acid), 360 mg of C. aurantium extract (6%), and 225 mcg of chromium polynicotinate) or a commercial decaffeinated coffee consumed 30 min before exercise. They reported no differences in peak power or mean power, fatigue index, or excess post-exercise oxygen consumption (EPOC). (See Table 2). Further, as explored in the section titled: Control issues in coffee research and practical application, Greer et al. [121] assessed the effects of anhydrous caffeine intake (6 mg/kg) on the performance of four 30-s Wingate sprints and did not find an ergogenic effect on power output. Exercise mode does not appear to change this lack of effect. In 2020, Alkatan and colleagues [19] dosed college-aged male swimmers with two shots of coffee containing 250 mg caffeine vs. a similar 0 mg decaffeinated beverage and did not observe improved sprint times in a 25-m simulated freestyle swimming competition. The metabolic energy system involved, population specificity, and/or the skill necessary to fully capitalize on ergogenic effects of caffeine may have been factors. More coffee-focused research on sprints is needed.

Experimental models of multiple set resistance exercise are another approach to examine muscle performance. Richardson and Clarke [44] compared the effect of ingesting a placebo, decaffeinated coffee (Nescafe 0.15 g/kg dissolved in 600 ml of hot water at 68.9 ± 2.5°C), and caffeine-dose-matched anhydrous caffeine (5 mg/kg), coffee (Nescafe 0.15 g/kg prepared identically), or decaffeinated coffee (0.15 g/kg) plus anhydrous caffeine (5 mg/kg) on benching and squatting (60% 1RM) in nine resistance-trained (>1 yr) adult males. Each session was completed within 30 min. At the start of each session, subjects were given 15 min to fully consume either the treatment beverage or capsules and water, after which time they rested for the remainder of the pre-exercise hour. The investigators reported significant differences in total weight lifted for the squat between the interventions with a greater amount lifted during decaffeinated coffee plus anhydrous caffeine compared with decaffeinated coffee, caffeine alone, and placebo conditions. Further, total weight lifted during the coffee condition was significantly greater than that lifted under placebo, but not during the decaffeinated coffee condition. No significant differences occurred during bench pressing. The investigators concluded that both coffee and decaffeinated coffee + caffeine improved resistance performance (Richardson and Clarke 2016). Collectively, these findings may also suggest an entourage effect, whereby the complex matrix of coffee confers some benefit to squat performance.

In 2019, Grgic and colleagues [133] performed an umbrella review of 21 meta-analyses on caffeine supplementation (mostly in young men) that included evidence on muscular strength, muscular endurance, and anaerobic power – as well as aerobic endurance (e.g. time to exhaustion) – and concluded that: 1) Caffeine was ergogenic in a broad range of exercise tasks and 2) Coffee was likely an effective ergogenic aid in resistance and sprint exercise when the caffeine dose falls between 3 and 6 mg/kg. These authors noted that coffee, per se, was relatively under-explored and that volume of ingestion is a practical consideration.

## 7. Effects of coffee on aerobic performance

A limited number of studies have investigated the effects of coffee on aerobic exercise performance. Most have reported an ergogenic effect [26,27,29,32,37,134,135] but some have not [2,41]; the specific methodology is important to consider when interpreting the evidence.

### 7.1. Running

As noted earlier, in 1998, Graham and colleagues [2] examined 9 adult (21–47 yr) trained endurance runners and dosed them with 7.15 ml/kg coffee (regular coffee, decaffeinated coffee + caffeine, decaffeinated coffee) 60 min before a run until exhaustion at 85% VO_2_max [2]. The various coffee conditions did not yield a difference in endurance time, but administration of anhydrous caffeine capsules did increased time to exhaustion. The authors [2] speculated that the ergogenic effects of caffeine may be altered by some other components in coffee. (See on History and on Control Issues in Coffee Research). However, other studies indicate that ingestion of caffeine (anhydrous or in coffee) can increase endurance, particularly in prolonged exercise lasting 30–120 min [134–136]. In shorter aerobic trials, Clark [26] concluded that 1-mile race time was 1.3% faster in trained male runners following the ingestion of caffeinated coffee compared to a decaffeinated variety and 1.9% faster compared to a placebo beverage. Similar performance benefits have also been depicted in healthy sedentary males [37] suggesting that training status may not be a limiting factor for performance benefits when consuming caffeinated coffee 60 min prior to single-bout aerobic exercise.

### 7.2. Cycling

Acutely consumed (i.e. pre-exercise) caffeinated coffee has been reported to enhance various aspects of endurance cycling in most, but not all studies. Clark [27] explored the effects of acutely ingesting 0.09 g/kg coffee containing 3 mg/kg of caffeine on 5 km cycling time in adult males and females. Coffee ingestion significantly improved performance times in both sexes by an average of 9 s and 6 s, respectively. Using a different type of acute aerobic protocol, Hoffman et al. [33] concluded that college-age males and females enhanced their time to exhaustion from 27.3 ± 10.7 min to 35.3 ± 15.2 min during an aerobic cycle test at 75% VO_2_max 30 min after consuming 1.5 cups (6.4 ± 1.2 g/kg caffeine) of a nutritionally enriched caffeinated coffee beverage (JavaFit) compared to a non-specified commercially available decaffeinated control. There was no unenhanced caffeinated coffee intervention. As noted earlier, per 1.5 cup serving, the nutritionally enhanced coffee contained 450 mg of caffeine, 1,200 mg of *Garcinia cambogia* (50% hydroxycitric acid), 360 mg of *Citris aurantium* extract (6%), and 225 mcg of chromium polynicotinate, making it difficult to attribute the benefits to coffee.

Hodgson et al. [32] observed trained cyclists (VO_2_max 58 ± 3 mL∙kg^−1^∙min^−1^) with low habitual caffeine intake (≤300 mg/day) for 30 min of steady state cycling at 55% VO_2_max followed by an energy-based time trial of 70% Wmax in the quickest time possible (approximately 45 min). Investigators compared 600 ml beverages consumed 60 min before exercise of 5 mg of anhydrous caffeine/kg, instant coffee (5 mg caffeine/kg), decaffeinated coffee, and a placebo containing 8 mg of quinine sulfate, a common ingredient found in tonic water that gives the beverage a bitter taste and imitates the coffee flavor, but does not initiate known ergogenic effects in small doses. Average aerobic power for caffeine and coffee during the time trial was significantly greater when compared to the decaffeinated coffee and placebo (294 ± 21, 291 ± 22, 276 ± 23 277 ± 14 watts, respectively). Similarly, performance times during the time trial were significantly faster (∼5.0%) for both caffeine and coffee when compared to the decaffeinated coffee and placebo (38.35 ± 1.53, 38.27 ± 1.80, 40.31 ± 1.22, 40.23 ± 1.98 min respectively). There were no significant differences in finishing time between the caffeinated coffee and anhydrous caffeine conditions, suggesting that coffee containing a moderately high caffeine dose is equally effective as anhydrous caffeine at improving endurance exercise performance.

Furthermore, Higgins et al. [98] conducted a review on the effects of pre-exercise caffeinated coffee as an ergogenic aid for endurance, and concluded that coffee providing 3.0–8.1 mg/kg (1.36–3.68 mg/lb) of caffeine administered ≥45 min before exercise may be used as a safe alternative to anhydrous caffeine to improve cycling and running performance and that coffee overall significantly reduced perceived exertion during testing.

In contrast to pre-exercise protocols, Nieman et al. (2018) [41] explored the effects of high- chlorogenic acid (CGA) coffee “loading” on a 50-km cycling time trial, postexercise inflammation, oxidative stress, and mood state. Male (36.1 ± 3.3 yr) and female (40.0 ± 3.3 yr) cyclists (*N* = 15) consumed 300 ml of either high CGA (1066 mg chlorogenic acid, 474 mg caffeine) or lower-dose (187 mg chlorogenic acid, 33 mg caffeine) Turkish brewed coffee every morning for two weeks. The 50-km cycling time performance (98.8 ± 3.2 v 99.2 ± 3.5 min, *p* = 0.71) and average power (169 ± 13.3 v 171 ± 13.3 W, *p* = 0.60), did not differ between trials. Higher ferric reducing ability of plasma (FRAP) levels were measured after exercise with high CGA coffee versus a lower-dose control. No differences between high CGA coffee and the control drink were found for postexercise increases in plasma IL-6 and hydroxyoctadecadienoic acids. Total mood disturbance (TMD) scores were lower with high CGA coffee versus control drink – an effect that did not differ between sexes. No significant differences were reported for average oxygen consumption, average respiratory rate or average rating of perceived exertion after an hour between the high CGA coffee and control trials. These findings suggest that the ergogenic effect of caffeinated coffee on cycling is limited to the acute setting, rather than with chronic dosing as is commonly seen with nutritional interventions such as carbohydrate loading.

## 8. Repeated bout (same day) power and endurance performance

While caffeinated coffee ingestion appears to be ergogenic for single-bout power and endurance tasks, less is understood about repeated same-day dosing. The differences in bioavailability and half-lives of various coffee compounds (e.g. polyphenols and caffeine) make coffee’s effects on repeated bouts potentially different than those of simple caffeine. See also the ISSN position paper specific to caffeine [59]. More research is needed.

## 9. Effects of coffee on training adaptations

Most studies exploring the effects of coffee ingestion have focused on the acute effects. Future studies are needed to assess the effects of long-term strategic use during training. For example, the effects of coffee may differ from anhydrous caffeine or other stimulants regarding glucose and glycogen metabolism, neuroendocrine activity, and blood flow [7,55,137–139]. This effect has led some researchers to use the term coffee “paradox” [32,140] and should be explored more specifically in athletic populations. Further, the question of a “super-training effect” should be explored; that is, does repeated use (and repeated acute ergogenesis) contribute to superior long-term adaptations? More research is required.

## 10. Effects of coffee on variables related to body composition

In addition to studies on ergogenic effects, a small body of literature has examined coffee’s effects on fat oxidation, body composition, and satiety. Early research by Costill, et al. [29] suggested increased fat mobilization and oxidation with caffeinated coffee ingestion. Although other researchers using various moderate-to-intense exercise methodologies (percent VO_2_max, caffeine vehicle, and dose, etc.) have not found significantly lower RER values after coffee or caffeine ingestion [24,108–110,112], other data suggest chlorogenic acids (329–600 mg/d over 5–28 days) may increase fat oxidation in non-exercise settings [50,141]. Limited data from human interventions (4–24 weeks) suggest a decrease in fat mass after administering coffee rich in chlorogenic acids [142–144]; this effect is based on animal data and may be due to interactions with gut microbiota [145]. Thus, application to athletes seeking body composition changes is not yet appropriate. Regarding muscle-specific effects, observational studies in middle-aged to older persons [146,147] suggest a positive relationship between coffee intake and skeletal muscle mass indices (anti-sarcopenic effects), but this result is supported by animal studies [148,149] and more work is needed with athletes of various ages. Lastly, researchers have investigated the effects of coffee on hunger and satiety with mixed results [18,150,151]. Application to weight management in exercisers will require more consistent findings. Taken together, the effects of coffee and its components on body composition and related variables are at times suggestive of benefits; however, further work is required in sports settings.

## 11. Side effects of coffee use

Being a complex matrix of compounds, most research into coffee’s side effects focus on caffeine, which is addressed in another ISSN position paper [59]. Still, there are issues to address relative to coffee, proper. First, there is controversy surrounding whether coffee is carcinogenic due to trace amounts of acrylamide or is anti-carcinogenic due to its overall matrix of compounds [152–154]. In the United States, according to California’s Safe Drinking Water and Toxic Enforcement Act of 1986 (“Proposition 65”), coffee would have carried a cancer warning due to the presence of acrylamide [155].

However following years of civil litigation on the issue [153], the California Office of Environmental Health Hazard Assessment (OEHHA) decided the dose in coffee was too low to be a carcinogenic risk [155].

In 2019, the FDA issued a statement in “strong” support: “ … [T]he California agency that administers Proposition 65 has proposed a regulation to exempt coffee from a Proposition 65 cancer warning. The FDA strongly supports this proposal. … We’ve taken this position because we too have carefully reviewed the most current research on coffee and cancer and it does not support a cancer warning for coffee. In fact, as our letter to California states, such a warning could mislead consumers to believe that drinking coffee could be dangerous to their health when it actually could provide health benefits. Misleading labeling on food violates the Federal Food, Drug, and Cosmetic Act. No state law can require food to bear a warning that violates federal law [156]. A related matter is the temperature at which hot coffee is consumed. Chronic consumption of hot to very hot beverages (subjectively determined) has been associated with esophageal cancer risk; although this is not unique to coffee, it may be a valid consideration when consuming it regularly at high temperatures [157].

A second controversy over side effects is whether the caffeine content of coffee carries a dehydration risk. (See section titled: Coffee absorption and transport). Maughan and colleagues [158] examined the impact of 13 different commonly consumed beverages, including coffee (212 mg of caffeine), on urine output and fluid balance. Recreationally active, healthy male subjects had similar urine output in the four-hour period following consumption of water and coffee. The acute effects of low (3 mg/kg or 260 ± 45 mg) and high (6 mg/kg or 537 ± 89 mg) levels of caffeine consumed via coffee on fluid balance in habitual coffee drinkers were investigated by Seal et al. [159]. They determined that a dose of 6 mg/kg of caffeine in coffee resulted in an acute diuretic effect, while the lower dose of 3 mg/kg was not different from water. Thus, the risk of dehydration may be dependent up on the caffeine content consumed.

Thirdly, anxiogenic effects can be a concern, particularly for athletes who endure the added stress of competition, depending on dose and individual differences. Anxiety exacerbation is likely due to the caffeine content of coffee. (See also information pertaining to sympathetic drive, below). For example, Watson and colleagues [130] reported higher “tense aspect of mood” in caffeine replete participants (200 mg twice daily for one week) compared to controls. This conclusion is not uncommon in the literature. According to Guest 2021: “Caffeine ingestion is also associated with increased anxiety; therefore, its ingestion before competition in athletes may exacerbate feelings of anxiety and negatively impact overall performance. Increased jitters, anxiety, and arousal associated with caffeine ingestion also needs to be considered within the specific demands of each sport … ” [59]. Further, compared to males, young females may experience increased jitters following regular coffee (100 mg caffeine) ingestion [125]. In some contrast, coffee, with a “low dose” of caffeine (100 mg caffeine), has been shown to have a greater arousal effect on males compared to females [160]. Adan et al. [160] also found that the decaffeinated beverage (decaffeinated coffee with 5 mg caffeine) increased arousal 10–30-min post consumption to a greater degree in females compared to male participants. Given potential metabolic (clearance) differences between the sexes, particularly with regard to oral contraceptives or estrogen replacement therapy [31,161–163], more research on mood and anxiety is needed. The impact of coffee polyphenols on the nervous system and on mood of both sexes should also be considered. Coffee contains dopaminergic components beyond caffeine [124,164]. Further, coffee’s polyphenolic components appear to be neuroprotective and to beneficially influence mood, including reduced anxiety scores and depression [30,165–168]

Fourth, there are contradictory data as to whether coffee improves, is ineffectual, or hampers glycemia and blood flow. Both research design and inherent coffee compounds come into play. Specifically, differences in observational studies versus intervention trials are an issue, as are differences regarding the oppositional effects of caffeine versus other coffee components [12,140,169–171]. From a glycemia and diabetes prevention perspective, Cornelius [172] emphasized a dose–response decline in Type 2 diabetes in an analysis of more than 25 prospective cohort studies across the United States, Europe, and Asia. Interestingly found in the same issue of that journal, a 24-week randomized controlled trial using four cups per day of instant coffee (Robusta with creamer), in overweight but healthy Asians showed no effect on insulin sensitivity or glucose metabolism [142]. Perhaps long-term mechanisms are at work that cannot be addressed in most intervention trials. Both study type and genetic predispositions may be reasons for such discrepancies. Population specificity beyond ethnicity further complicates conclusions. For example, exercisers are known to exhibit improved blood glucose regulation compared to sedentary persons. (Also see section titled: Coffee and exercise recovery). A comparison of cross-sectional studies versus acute intervention trials among athletes could shed light on the glycemia issue. Regarding blood flow, Higashi [140] described the interaction between caffeine and other coffee components as a “coffee paradox,” citing conflicting literature and relevant control issues, such as choice of endothelial function test and chlorogenic acid content.

Coffee is also paradoxical regarding its effects on the nervous system. For example, coffee is known to increase epinephrine and related metabolite concentrations [2,52,124]; however, rather than solely exhibiting increased sympathetic drive, habituated and/or physically active consumers of coffee components (caffeine and chlorogenic acids) have been shown to exhibit tendencies toward reduced mental stress [173] and neutral-to-beneficial effects on heart rate variability (HRV) compared to baseline or to coffee-naïve controls [139]. This lack of harm to the autonomic nervous system is not uncommon in the literature. Although the measurement of HRV has several parameters, an increase is generally considered to be beneficial and indicative of greater parasympathetic drive (i.e. a “restive-digestive” state more conducive to recovery). In partial contrast, and again focusing on habituation, Zimmermann-Viehoff, et al. [174] reported increased HRV after healthy caffeine-naïve participants drank decaffeinated espresso and a blunted HRV increase in healthy, but caffeine habituated subjects who drank the decaffeinated espresso beverage. Athletes and caffeine intake have also been studied using HRV. Karayigit and colleagues [35] examined the effects of 3 mg/kg and 6 mg/kg caffeine from coffee on caffeine-naive female athletes (rugby, handball, and soccer) and reported no adverse effects on HRV parameters along with improved lower body muscular endurance and cognitive performance [35]. This is in some contrast to studies using caffeine alone, in which acute parasympathetic recovery was delayed within the hour after aerobic and strength exercise [175,176]. Timing of measurement may be a factor. Whether any differential effect of coffee-versus-caffeine exists for naïve or habituated coffee drinkers, and whether this makes a practical difference when used as a pre-exercise stimulant, remains a question.

Sixth, coffee has a history of equivocal findings regarding cardiovascular effects according to Chrysant [177]. As is common with other effects from coffee, discrepancies are typically dependent on dose, bean type, brew method, and individual sensitivity. The once-held conclusion of increased risk of cardiovascular disease has been reversed by several more recent prospective cohort studies and meta-analyses, again according to Chrysant [177]. Further, according to Mattioli (2014) [178] there is a protective effect on cardiac arrythmias with a moderate intake of caffeine, equivalent to that found in one to four cups of coffee/day. The source of the caffeine, whether it be from coffee or from another source, impacts this effect [178]. Still, sensitive persons or those with a family history of supraventricular arrhythmias should exercise caution with regard to the caffeine dose. Further, a recent review by Barrea and colleagues (2021) [7], concluded timing and drug interactions should also be taken into account. Regarding exercise, caffeine ingestion typically has a small or inconsistent impact on heart rate, although high-chlorogenic acid coffee may increase heart rate [41,59]. Similarly, regarding caffeine and resistance exercise, mixed findings suggest increases in (typically systolic) blood pressure [133]. According to Guest, et al. [59], for those with the CYP1A2 AA genotype, the inclusion of regular physical activity appears to negate the increase in blood pressure and anxiety resulting from caffeine ingestion [59].

Lastly, the effects of coffee on sleep architecture have been studied, with early research on healthy adults suggesting similar dose–response disturbances between caffeinated coffee (one to four cups) and caffeine 30 min prior to bedtime [179], with more recent research examining genetic differences [180] and the potential benefits of chlorogenic acid (600 mg × 5d), on sleep latency [141]. An evidence-based practical approach would include self-monitoring of sleep disturbances, limit caffeinated coffee intake to the morning or afternoon hours, and to consider decaffeinated coffee late in the day. Finally, a related issue is whether caffeinated coffee is beneficial in a sleep-deprived state. According to Guest et al. (2021), caffeine may improve cognitive and physical performance in some individuals under conditions of sleep deprivation, though the effect is not indefinite. Further, Chaudhary et al. [181] studied sleep deprivation among military personnel and concluded that caffeinated drinks (primarily coffee) “improved their cognitive and behavioral outcomes and physical performance.”

Taken together, the above potential side effects of coffee – with the exception of dehydration – require further investigation in different populations, and under varying exercise-related conditions.

## 12. Control issues in coffee research and practical application

### 12.1. Placebo effects, blinding, types of controls (water, decaffeinated coffee, etc.)

A placebo is an inert treatment [182], and thus its effect on physical performance is due to a belief that a beneficial substance has been received [183]. Such a placebo effect is prevalent in sport, with one-third of athletes reporting better performance than anticipated due to false information [184]. The placebo effect of caffeine was demonstrated with well-trained cyclists that improved anaerobic performance when they believed they had ingested a high dose (9 mg/kg) and a lower dose (4.5 mg/kg) of caffeine compared to a placebo, when in reality they had received a placebo in every trial [183]. Ergogenic outcomes were also realized in a study by Foad et al. [185] with a group of competitive endurance cyclists who were told they ingested caffeine, but had actually been given a placebo.

The selection of an appropriate placebo is important in designing a valid study. The majority of researchers have used decaffeinated coffee of a similar roast as the placebo beverage [2,20,21,24,26,32,35,38,44,99,102,186]. Typically, the same brand, roast, and preparation methods have been used for the coffee treatment and the decaffeinated placebo. However, subjects have been reported to be able to correctly identify the coffee treatment between 56% [20] and 72% [2] of the time.

A closer look at one of the landmark investigations into coffee is warranted. (See section titled: History of research on coffee related to exercise). To examine the possible antagonism of compounds in coffee, Graham et al. [2] provided subjects with 4.45 mg/kg caffeine in capsules and coffee. Placebos used were dextrose capsules and decaffeinated coffee. Similar increases in plasma methylxanthines were found in the caffeine capsules and coffee trials. Endurance performance was significantly improved only in the caffeine capsule trial. Graham et al. [2] speculated that the ergogenic effects of caffeine may be altered by some other components in coffee. For example, with commercially available coffees, variations in roasting processes may affect the concentration of chlorogenic acids. Mills et al. (2013) [187] documented a strong inverse relationship between roasting time and the chlorogenic acid content. Thus, the selection of type and roast of coffee may have a sizable impact on the amount of chlorogenic acids ingested. Such factors may impact exercise performance [187]. As the chlorogenic acid content may negatively impact the ergogenic effects of caffeine, Pickering and Grgic [188] have recommended the use of decaffeinated coffee and also a beverage that tastes similar to coffee, but lacks caffeine and other nutritional properties. A few researchers have used this model [25–27,32]. Other researchers have used a coffee alternative or flavoring [25–27,32,51,102], and yet others have used no control treatment or group [108,189]. Lack of consistent control (placebo) conditions make it difficult to assess the direct benefits of various coffees and their constituents. Thus, sweeping generalizations regarding “coffee” should be avoided.

### 12.2. Timing and duration of ingestion

Following coffee consumption, time to peak saliva caffeine concentration is 42 min which is faster than caffeine consumed via capsule form [77]. Maximal concentrations of caffeine are found in plasma within 30–60 min, regardless of the amount ingested [114,190,191]. Following ingestion of two to three cups of coffee, the plasma levels of caffeine reach 20–50 µM [190]. Keeping in line with this timing, the majority of researchers have utilized protocols where coffee was ingested 60 min prior to exercise/performance measure [2,20,21,24,26,28,32,35,38,99,102,186], with fewer using ingestion times of 45 min [25,44] and 30 min prior to exercise [33,41].

In addition to the variation in time to complete consumption prior to exercise or testing, coffee ingestion time itself has not been standardized. Some protocols have used ingestion times of 2–5 min [26,38], 10–15 min [2,27,28,33,35,44], no specified times provided but they were replicated with each trial [20,21], and no time specified at all [24,25,32,41,51,99,102,186,189]. The lack of control for timing of ingestion may impact the time at which caffeine, and other bioactive compounds, are at maximal concentrations in the plasma and thus may impact the ergogenic potential of coffee.

### 12.3. Sex-specific effects of coffee on exercise performance

The majority of studies done on coffee and exercise performance have been conducted on only male subjects [25,26,32,38,44,51,99,186]. A number of researchers have included both males and females, without examining differences in performance between the sexes following coffee ingestion [2,20,21,24,26,28,33,102]. Clarke & Richardson [28] tested 46 recreationally active participants, 19 of which were female that had been using monophasic oral contraceptives for at least 3 months prior to the study. Testing was conducted on days 5–8 and 19–22 of the menstrual cycle as energy metabolism is not affected at these times [192]. Similar improvements were seen for males and females in the coffee trials compared to the placebo.

Very few studies have been conducted comparing the effects of coffee ingestion on exercise performance of males and females. Pickering & Grgic [162] speculated on the reason for the lack of research in this area stating that females are a more physiologic complex cohort, and that oral contraceptive and menstrual cycle stages can alter caffeine metabolism speeds. The half-life of caffeine has been shown to be almost doubled for women on oral contraceptives, mainly in the latter half of the luteal phase [161]. However, McLean and Graham [193] demonstrated no identifiable sex differences in any caffeine pharmacokinetic measures between males and eumenorrheic females not on oral contraceptives. As noted earlier, Nieman et al. [41] examined the impact of two weeks of high chlorogenic acid (CGA) coffee consumption on mood state and performance of a 50-km cycling time trial. Following the supplementation period with either the high chlorogenic acid coffee or placebo, 10 males and 5 females ingested 300 ml of CGA coffee (474 mg caffeine and 1066 mg CGA) or decaffeinated coffee (33 mg caffeine and 187 mg CGA) 30 min prior to the 50-km cycling time trial. As there was no difference in plasma caffeine levels between males and females, their performance data were combined [41]. Clarke et al. [27] provided 19 males and 19 females with coffee (3 mg/kg) prior to a 5-km cycling time trial. Similar increases were found in salivary caffeine levels between both sexes. Although males attained higher power outputs than females, when adjustments were made for baseline performance, no significant difference in improved performance following ingestion of coffee were seen between the sexes [27].

Mielgo-Ayuso et al. [194] conducted a systematic review on the effect of caffeine supplementation on sports performance based on differences between sexes. A variety of caffeine sources were included in the review, including coffee, commercial drinks, anhydrous caffeine mixed with water, and caffeinated gum. Sex differences in fatigability notwithstanding, no differences between sexes and caffeine supplementation were demonstrated with regard to aerobic performance or fatigue index [194]. Given that females are on average of lower mass than males, this could impact their conclusions. However, due to small sample sizes and limited number of studies available, it is difficult to generalize the recommendations. Mielgo-Ayuso et al. [194] recommend considering the menstrual cycle when examining the ergogenic potential of caffeine.

### 12.4. Habituation, coffee, and exercise performance

As was stated earlier, caffeine acts to antagonize adenosine A1 and A2A receptors, and with regular caffeine consumption there is an upregulation of the number of these receptors [195]. Thus, it has been deduced that habitual consumption of coffee, and other caffeinated products, may influence the dose of caffeine necessary to elicit an ergogenic effect [196,197]. In fact, Sökmen et al. [197] proposed dose response curves for non-users, average caffeine users (<3 mg/kg), and heavy caffeine users (>6 mg/kg). The ranges for ergogenic effectiveness recommended are 2–5 mg/kg for non-users, 3–6 mg/kg for average users, and 7–10 mg/kg for heavy caffeine users. They suggested that the dose response curves are shifted to the right and that the ergogenic effectiveness is attenuated with increased caffeine intake [197]. Based on these theories, researchers have utilized abstinence periods in an effort to eliminate the impact of habituation. For studies performed with coffee, the most frequently used abstinence periods have been the day of the trial [24,33], such as 12 h [26–28,44], 18 h [20,21], 24 h [32,35], and 48 h [2,38,51] prior to the trial.

One consideration that must be taken when requiring subjects to abstain from caffeine is the potential of withdrawal symptoms that may impact performance measures. Withdrawal symptoms peak at 24 to 48 h following cessation of caffeine consumption, and these effects return to baseline in 4 to 7 days [197]. However, some individuals may experience these symptoms as early as 3–6 h after caffeine cessation. They may also have symptoms persist for as long as one week [198,199]. Given that most of the abstinence periods fall in the window of peak withdrawal symptoms, it is possible that subjects are beginning experimental trials with headaches or other symptoms that may mask the effects of coffee. An additional consideration for researchers when designing studies accounting for caffeine withdrawal is the reality that many athletes do not abstain from caffeine for several hours-days prior to their routine training and/or competition.

Collomp et al. [200] examined the influence of regular intake of coffee on the pharmacokinetic parameters of caffeine. Subjects included six heavy coffee drinkers (four to five cups of coffee/day) and six light coffee drinkers (<1 cup of coffee/day). Following consumption of 250 mg of caffeine, the heavy coffee drinkers had a greater half-life elimination and volume of distribution than the light coffee drinkers [200]. Clarke and Richardson [28] investigated the influence of habitual caffeine intake on 5-km cycling time trial performance in 46 recreationally active participants (27 males, 19 females), composed of 16 high (>6 mg/kg/d) caffeine consumers and 30 low (<3 mg/kg/d) caffeine consumers. A 12-h caffeine abstinence period was used. The 5-km cycling time trial performance was improved in both the low and high caffeine consumers [28]. In a recent systematic review and meta-analysis, Carvalho et al. [201] determined that habitual caffeine use did not affect the ergogenic effect of caffeine in trained and untrained male and female participants.

### 12.5. Training status, coffee, and exercise performance

A few studies on coffee and exercise have been done with relatively untrained individuals [25,99,189] and recreationally active subjects [24,27,28,44,108], with the majority of researchers focusing on sport-specific trained athletes [2,20,21,26,32,33,35,38,41,44,51,102,186]. In examining the effectiveness of coffee for exercise performance, the training status of participants may be a significant factor, particularly for high intensity performance [202]. Anaerobic power is frequently assessed using the Wingate test for physically active individuals. Typically, the test is performed by recreationally active, sport athletes (football, rugby, basketball, etc.), or resistance trained individuals. Greer et al. [121] assessed the effects of caffeine intake on the performance of four 30-s Wingate sprints separated by 4 min. They did not find an ergogenic effect of caffeine on power output. However, Greer et al. [121] stated that although the participants were physically active, “none [were] accustomed to the intense exercise experienced in this study.” According to their findings, it is unlikely that complete motor unit recruitment occurred, as caffeine’s effects in untrained individuals are primarily seen in fiber type(s) utilized during training [121]. That is, motor unit adaptations may play a role in the extent to which caffeine exerts effects. In a random double-blind placebo-controlled study, Collomp et al. [203] administered 250 mg of caffeine to seven regional competitive swimmers and seven former members of a swimming club to examine the impact of specific training on the effectiveness of caffeine. The dose of caffeine was consumed 60 min prior to performing two 100-m freestyle sprints interspersed with a 20-min rest period. Swimming velocity was significantly higher with caffeine for only the training swimmers in both the first and second 100 m swims. The researchers concluded that adaptations resulting from specific training appear to be necessary to benefit from caffeine in sprint performance [203]. This lack of specificity of training may also apply to populations tested in much of the research published on caffeine’s effects on resistance training performance. Researchers routinely study “physically active,” “resistance trained,” or team sports athletes as subjects. However, the definition of resistance trained has not been consistent among authors. Some have listed training histories as few as eight weeks to as much as 12 years of training or more. As stated earlier, it is likely that motor unit activation is less complete for lesser trained individuals [121] and thus researchers may not be able to adequately assess caffeine’s impact on performance with these subjects. Thus, a true ergogenic benefit may only be observed with trained participants. This may be due to the higher muscle mass and concentration of adenosine receptors in trained athletes [26,204]. Additionally, elite athletes are more reliable at performance and have a smaller coefficient of variation, which may allow for assessment of accurate effects of an intervention [205].

### 12.6. Genetics, coffee, and exercise performance

Interindividual differences in response to coffee or caffeine have been suggested as a reason for the lack of statistically significant improvements in performance seen in studies [2,20,21,24]. Church et al. [24] identified 60% of their subjects as responders to coffee (faster time trial), with an equal number of males and females in this group. The responders cycled on average 5% faster in a 5-km time trial time, with the greatest difference of nearly 300 s faster following coffee (3 mg/kg caffeine) ingestion vs decaffeinated coffee, while the most dramatic non-responder cycled nearly 140-s slower in the coffee condition [24]. Similarly, Anderson et al. [20] found 56% of their subjects achieved a higher peak power, and 22% achieved a lower peak power output, during a 30-s Wingate cycling test during the coffee trial (280 mg; 3–6 mg/kg caffeine) compared to the decaffeinated coffee trial. These values are similar to the estimates made by others that 47–53% [206] to as high as 64% [126] of subjects are caffeine responders.

The interindividual differences in response to coffee or caffeine appear to be due to variations in the CYP1A2 gene [207]. This gene encodes the CYP1A2 enzyme which acts to metabolize over 95% of caffeine [208]. Individuals with the CYP1A2 AC or CC genotype are slow metabolizers of caffeine, while those with the AA genotype are fast metabolizers of caffeine. Guest et al. [207] examined whether variations of the CY1A2 gene affected low (2 mg/kg) and moderate (4 mg/kg) doses of caffeine on endurance performance. Of 101 competitive male athletes, 51.5% were identified as slow metabolizers and 48.5% were fast metabolizers. Caffeine at doses of 2 and 4 mg/kg improved endurance performance in only the fast caffeine metabolizers (those with the CYP1A2 AA genotype). Additionally, both caffeine doses (2 and 4 mg/kg) had no impact on performance for slow metabolizers of caffeine with the AC genotype while performance was impaired for those with the CC genotype [207]. Note: As mentioned earlier in the paper, coffee has constituents that may affect the physiological effects of caffeine. More coffee-specific research is needed.

## 13. Coffee and exercise recovery

Recovery from exercise includes several factors, including water and substrate repletion, repair of muscle microtrauma and declining inflammation, and a return to a neuro-endocrine state that is less sympathetically driven. Coffee’s complex nature has led exercise physiologists to consider it as both a pre-exercise stimulant and an antioxidant-rich recovery aid, as described throughout this review. To recap, researchers to date have demonstrated hydration effects similar to water [83] enhanced glycogen recovery [137], and antioxidant effects in the general healthy population [5,9,209]. Whether these antioxidant effects extend to tissue recovery among athletes is yet to be determined. No definitive conclusions from human studies are known regarding the specific effects of coffee on delayed-onset muscle soreness (DOMS), although sustained caffeine ingestion may decrease it [210]. Further, Dirks-Naylor [148] reported that coffee may stimulate regeneration of injured muscle in animal and *in vitro* models. On the other hand, in observational studies, data are mixed regarding correlations with the inflammatory marker C-reactive protein (CRP) [211,212]. Regarding mental stress and the autonomic nervous system, neutral-to-positive effects have been reported that may be different for coffee-habituated participants [139,173,174,213]. For details on autonomic nervous system effects, including those regarding caffeine versus coffee, see the section titled: Side effects of coffee use. Lastly, there are mixed findings on cortisol production in healthy non-athletes [150,214–216]. Any risk or benefit in this regard could depend on the presence and type of overtraining/under-recovery [217].

## 14. Optimal protocols of coffee ingestion related to exercise

Optimal protocols for using coffee for acute ergogenic effects, and perhaps recovery, are closely tied to the control issues noted in the section titled: Control issues in coffee research and practical application. These include dose of caffeine and total chlorogenic acids (which are affected by many factors in sourcing and preparation), timing, and awareness of individual sensitivity to coffee:

1. Dose: Consumption of two to four cups (approximately 473–946 ml or 16–32 oz.) of typical medium-roast hot-brewed or reconstituted Arabica instant coffee appears optimal, depending on individual sensitivity, habituation, and body size, providing a caffeine equivalent of 3–6 mg/kg (among other components such as chlorogenic acids at approximately 100–400 mg per cup). The upper end of the range could pose practical issues regarding volume of liquid, and individuals weighing over 100 kg would need still more. Intake of more concentrated coffees, such as espresso, instant/micro-ground varieties, Robusta bean or light roast, or cold-brewed coffee, would be reduced. Consumers should seek caffeine information per serving and consider the relative caffeine dose of 3–6 mg/kg, and perhaps 800 mg of total chlorogenic acids. More research on chlorogenic acids and other polyphenols is needed.

- Timing (pre-exercise, acute): Keeping in mind a 15–30-min consumption period, the final sip should be consumed approximately 60 min prior to exercise. Individuals sensitive to caffeine or experiencing sleep disturbances may have to downward-adjust the dose during times of evening training.
- Genetic/individual tolerance: The heterogeneity of coffee as a food item is compounded by individual genetic and potential gut microbiota differences. Habituation may also be an issue, as are the intakes of other foods and drugs. Exercisers interested in coffee’s benefits should consult with a knowledgeable healthcare practitioner and consider the low end of the dosing range (two cups or 473 ml).

## 15. Interaction of coffee with other ergogenic aids

Studies show that many athletes who supplement with ergogenic aids often use a variety of products concomitantly [218–220]. In response, coffee is sometimes sold in combination with other sports-related nutrients. Examples include medium-chain triacylglycerols (MCT), protein, amino acids, probiotics, additional caffeine, botanicals, and creatine (although its stability in solution has been questioned, depending on the creatine type). This creates challenges for athletes. Much of the evidence used to justify usage of a supplement is derived from research done on that supplement in isolation of other products. However, because foods and supplements may act synergistically or antagonistically, the justification is insufficient to ensure maximization of performance gains. Several studies have evaluated coffee’s or caffeine’s effectiveness on performance enhancement when used in combination with other popular ergogenic aids such as creatine monohydrate and anhydrous caffeine. See Table 3. Data on a multi-ingredient “nutritionally-enhanced” coffee is described earlier in this position stand. Further, early work by Vandenberghe and colleagues [221] suggested that caffeine supplementation in combination with creatine ultimately eliminated the ergogenic effects of creatine during intense intermittent exercise. A counteracting effect on Ca2+ clearance was thought to dampen creatine’s ergogenic properties [221,222]. Other studies, however, have shown that it is possible to increase exercise performance through the administration of creatine along with coffee during maximal, high-intensity exercises [223,224]. As previously discussed, coffee appears to exert much of its ergogenic effects by antagonizing adenosine A1 and A2A receptors [87–92,94]. This major mechanism of coffee erogenicity appears to work mostly independently of creatine. Trexler and colleagues [51] sought to determine how creatine loading in combination with caffeine anhydrous or coffee would affect upper body strength, lower body strength, and sprint performance. A repeated sprint protocol was used in the laboratory to measure peak power and total work, as well as bench press and leg press 1RM and repetitions to failure. This exercise protocol was completed once before supplementation and once after. In the five days preceding the second round of exercise testing, the subjects began supplementing either with creatine (20 g/d), creatine and caffeine (20 g/d +300 mg/d), or creatine and instant coffee (20 g/d +8.9 g/d instant coffee (303 mg caffeine) for five days. Both bench press and leg press 1RM and repetitions to failure improved in all groups, except for one instance within the creatine and coffee group where the bench press repetitions to failure did not change with treatment. Sprint outcomes, however, did not significantly improve across all groups. Overall, this study showed no statistically significant impairment of anaerobic performance when supplementing creatine and instant coffee simultaneously.

It is also popular for ready-to-drink coffee beverages to have added caffeine beyond what naturally occurs in coffee to reach a greater total caffeine dosage [225]. In the United States, functional coffees and “shots” from brands like Bang, Monster, Starbucks, Stok, and others are capitalizing on added caffeine and blurring the coffee-energy drink market. One advantage could be reduced volume of intake, but due to the difficulty in knowing the precise dose of caffeine in some products, some sport dietitians may only use anhydrous caffeine with their athletes [59]. Details are included in a previous JISSN position stand that focused on energy drinks [226].

Other potential nutritional ergogenic ingredients, when co-ingested as part of coffee beverages, need further study. Not all nutrient combinations are synergistic nor even additive, as noted below. However, for habitual coffee consumers, potential does exist for increasing overall intake of desired nutrients.

## 16. Coffee-nutrient interactions

As previously mentioned, coffee is a complex matrix of numerous compounds [1–6]. Although caffeine plays a large role in coffee’s performance enhancing benefits, researchers throughout this review have pointed to the other inherent compounds as responsible for significant physiological effects. Considering that coffee contains many such compounds that could have such an impact, it would be wise to take into consideration the interactions between phenolic compounds in coffee and the vitamins, minerals, and phenolics in other foods [227]. The overall polyphenol content of instant coffee, for example, reduces iron absorption [228–230] in some studies by 60–90% [229]. This is because phenolic compounds, including chlorogenic acid, have aromatic ring structures with hydroxylic groups. The presence of hydroxyl groups can interfere with absorption within the intestinal lumen by forming complexes with metal cations, such as iron [231]. One corrective approach may be to co-ingest coffee with additives, but Hurrell et al. (1999) [229] did not find the addition of milk to coffee to improve iron absorption from a bread meal. Some professionals recommend separating intake of coffee from intake of iron supplements or iron-containing foods by at least 1 h [232]. In athletes suffering from performance detriments caused by iron-deficiency, it might be advantageous to reduce or remove coffee intake surrounding mealtimes.

The potentially negative effects of coffee on serum vitamin D action have also been reviewed, but practical applications remain unresolved. Belayneh and Molla [233] noted that coffee can interfere with osteoblast vitamin D receptors, thus limiting the amount of vitamin D that can be absorbed. Further, although controversial, some researchers have shown that caffeine consumed in coffee negatively affects calcium balance [234–237]. There is a dearth of research on how this negative affect on calcium balance from coffee goes on to affect bone mineral density in an athletic population, but an observational study of non-athletes showed that older women who consumed more than two cups of coffee per day over 12 years had a 69% higher fracture rate than those who did not consume coffee [233]. Although this is likely in part mediated by coffee’s effect on vitamin D physiology, there are other possible mechanisms by which caffeine and coffee potentially alter calcium balance. It could be an absorptive interference in the intestinal lining directly affecting the transport system for calcium. However, it could also be in part mediated by decreased serum inositol, a calcium metabolism regulator [238]. Despite there being multiple potential mechanisms by which coffee could negatively impact calcium balance, more research needs to be done on athletes. It is important to take vitamin D-calcium-bone factors into consideration when considering coffee as a supplement in this population. It is known to be a group with unique bone mineral issues, both positive (e.g. resistance training) and negative (e.g. female athlete triad).

## 17. Coffee and drug interactions

The research on drug – coffee interactions is incredibly complex. The varying compounds that make up coffee may individually alter absorption, distribution, and elimination of drugs in different ways. Furthermore, the hundreds of compounds that make up coffee can differ based on coffee varietal, environment, processing, and brewing method [239]. Therefore, this section will not serve as a comprehensive account to address all coffee – drug interactions. Rather, it will focus on interactions between coffee and drugs deemed most significant to exercise performance. Tcholl and colleagues [240] analyzed 3,887 doping control forms taken during 12 International Association of Athletics Federations World Championships and one out-of-competition season in track and field. Forms were submitted from athletes of all ages and from multiple continents. Some of the top reported medications used included nonsteroidal anti-inflammatory drugs (NSAID), β2-agonists, and contraceptive drugs. Aspirin is one NSAID that has been investigated for its interaction with coffee. The rate of aspirin absorption rapidly increased when 650 mg of aspirin was consumed along with two cups of coffee, equivalent to 120 mg caffeine [241]. An increase in gastric acid secretion is the mechanism of increased aspirin absorption caused by caffeine in coffee. No known research has been evaluated in humans for the interaction between coffee and β2-agonists, although both are known to be stimulant in nature. Regarding oral contraceptives, their use results in a 40% reduction of serum caffeine clearance [242]. It was proposed that three to four cups of coffee/day could result in a caffeine accumulation in females on oral contraceptives because of the interference of the treatment with CYP1A2 (see sections titled: Side effects of coffee use and Control issues in coffee research and practical application).

**Table 3.** Summary of studies exploring the effects of coffee in combination with other supplements.

Study	Participants	Exercise-related performance test	Coffee dose	Timing of ingestion	Additional supplement	Main findings
Trexler et al, 2016 [51]	54 resistance trained males (2.1 ± 2.1 yr)	Leg and bench press 1RM, 80% 1RM RTF, 5 10 s cycle ergometer sprints (1 min rest)	8.9 g instant coffee (303 mg caffeine)	Everyday for 5 days preceding the second round of testing	20 g creatine monohydrate	LP 1 RM ↑ LP RTF ↔ BP 1RM ↔ BP RTF ↔ Sprint PP ↔ Sprint TW ↔ Repeated Sprint fatigue ↓
Baumeister et al, 2021 [243]	7 young healthy adults	Capillary blood glucose and βHB; salivary caffeine every 40 min (total 240 min)	10 interventions with 10.4g DCF coffee powder in 250 ml water + 150 mg caffeine and varying amounts of coconut oil, MCT	Samples were taken post ingestion for 240 min	coconut oil + MCT	Glucose ↔ Plasma caffeine ↔ (among caffeinated interventions) βHB (CO-) ↔ βHB (CO+) ↑ βHB(CTL-) ↔ βHB (CTL+) ↑
Mills et al, 2017 [244]	15 males (26.3 ± 1.6 yr)	Flow-mediated dilation	3.6 g of ground coffee to 50 ml of hot water (110 mg caffeine)	Measures were taken before and 1, 3, and 5 hours post ingestion	Low polyphenol coffee (LPC) + 89 mg CGA per 3.6 g of coffee and a high polyphenol coffee (HPC) 310 mg CGA per 3.6 g coffee	Vascular function ↑ (LPC and HPC)
Roberts et al, 2007 [245]	10 recreationally active males (27.6 ± 4.2 yr) and females(29 ± 4.6 yr)	Modified Bruce VO2max test with RPE and Wingate test	1.5 cups (354 ml) of JavaFit™ Energy Extreme coffee (450 mg caffeine) or 6.5 g Folgers Regular brewed with 354 ml water (200 mg caffeine)	Measures were taken the last 20 minutes of each hour for 3 hours post-ingestion	1200 mg garcinia cambogia, 360 citrus aurantium, 225 mcg chromium polynicotinate	Peak VO_2_ ↔ VO_2_ at 3 min ↑ VO_2_ 10 min ↔ Post-exercise ↔ Time-to-exhaustion ↔ Maximal RPE ↔ Peak power ↔ Time to peak power ↔
McAllister 2020 [246]	10 university males	βHB, glucose, triglycerides (TAG), insulin, advanced oxidation protein products (AOPP), glutathione (GSH), malondialdehyde (MDA), hydrogen peroxide (H_2_O_2_)	16 oz. Folgers medium roast, 50 g ground coffee	0, 2, 4 h	0, 28, 42 g MCT	βHB ↑ TAG ↓ MDA ↓ (42g) H_2_O_2_ ↓ AOPP ↑ (42g) Insulin ↓

↑= significant increase; ↓= significant decrease; ↔ = no significant difference; 1RM = one repetition maximum; AOPP = advanced oxidation protein products; βHB = beta hydroxybutyrate;BP = blood pressure; CO+ = coconut oil present; CO- = coconut oil absent; CTL = control; GSH = glutathione; HPC = high-polyphenol coffee; LP = leg press; LPC = low-polyphenol coffee;MCT = medium-chain triglyceride; MDA = malondialdehyde; PP = peak power; RTF = repetitions to failure; TAG = triacylglycerol; TW = total work.

## 18. Position of the International Society of Sports Nutrition (ISSN)

Based on a review of peer-reviewed literature and critical analysis thereof regarding the effects of coffee on exercise performance, conducted by experts in the field and selected members of the International Society of Sports Nutrition (ISSN), the following conclusions represent the official Position of the Society:

1. Coffee is a complex matrix of hundreds of compounds. These are consumed with broad variability based upon serving size, bean type (e.g. common Arabica vs. Robusta), and brew method (water temperature, roasting method, grind size, time, and equipment). This affects consensus in the literature.
2. Coffee’s constituents, including but not limited to caffeine, have neuromuscular, antioxidant, endocrine, cognitive, and probable metabolic (e.g. glucose disposal, vasodilation) effects that impact exercise performance and recovery.
3. Coffee’s physiologic effects are influenced by dose, timing, habituation to a small degree (to coffee or caffeine), nutrigenetics, and potentially by gut microbiota differences, sex, and training status.
4. Coffee and/or its components improve performance across a temporal range of activities from reaction time, through brief power exercises, and into the aerobic time frame in most but not all studies. These broad (and varied) effects have been demonstrated in males (mostly) and in females, with effects that can differ from caffeine ingestion, per se. More research is needed.
5. Optimal dosing and timing is approximately two to four cups (approximately 473–946 ml or 16-32 oz.) of typical hot-brewed or reconstituted instant coffee, depending on individual sensitivity and body size, providing a caffeine equivalent of 3–6 mg/kg (among other components such as chlorogenic acids at approximately 100–400 mg/cup) 60 minutes prior to exercise.
6. Coffee has a history of controversy regarding side effects but is generally considered safe and beneficial for healthy, exercising individuals in the suggested dose range above.
7. Coffee can serve as a vehicle for other dietary supplements and it can interact with nutrients in other foods.
8. A dearth of literature exists examining coffee-specific ergogenic and recovery effects, as well as variability in the operational definition of “coffee,” making conclusions more challenging than when examining caffeine in its many other forms of delivery (capsules, energy drinks, “pre-workout” powders, gum, etc.).
